# Cell Wall Trapping of Autocrine Peptides for Human G-Protein-Coupled Receptors on the Yeast Cell Surface

**DOI:** 10.1371/journal.pone.0037136

**Published:** 2012-05-18

**Authors:** Jun Ishii, Nobuo Yoshimoto, Kenji Tatematsu, Shun’ichi Kuroda, Chiaki Ogino, Hideki Fukuda, Akihiko Kondo

**Affiliations:** 1 Organization of Advanced Science and Technology, Kobe University, 1-1 Rokkodai, Nada, Kobe, Japan; 2 Department of Structural Molecular Biology, Institute of Scientific and Industrial Research, Osaka University, 8-1 Mihogaoka, Ibaraki, Osaka, Japan; 3 Graduate School of Bioagricultural Sciences, Nagoya University, Furo, Chikusa, Nagoya, Japan; 4 Department of Chemical Science and Engineering, Graduate School of Engineering, Kobe University, 1-1 Rokkodai, Nada, Kobe, Japan; University of Connecticut, United States of America

## Abstract

G-protein-coupled receptors (GPCRs) regulate a wide variety of physiological processes and are important pharmaceutical targets for drug discovery. Here, we describe a unique concept based on yeast cell-surface display technology to selectively track eligible peptides with agonistic activity for human GPCRs (Cell Wall Trapping of Autocrine Peptides (CWTrAP) strategy). In our strategy, individual recombinant yeast cells are able to report autocrine-positive activity for human GPCRs by expressing a candidate peptide fused to an anchoring motif. Following expression and activation, yeast cells trap autocrine peptides onto their cell walls. Because captured peptides are incapable of diffusion, they have no impact on surrounding yeast cells that express the target human GPCR and non-signaling peptides. Therefore, individual yeast cells can assemble the autonomous signaling complex and allow single-cell screening of a yeast population. Our strategy may be applied to identify eligible peptides with agonistic activity for target human GPCRs.

## Introduction

G-protein-coupled receptors (GPCRs) constitute a large superfamily of cell surface receptors [1]. In humans, these 7-transmembrane proteins respond to external stimuli to regulate various cellular processes including taste, smell, vision, heart rate, blood pressure, neurotransmission and cell growth [2]. All members of the guanine nucleotide binding protein family (G-proteins) share a common mechanism for signal transmission following GPCR-agonist binding [3]. This universal signaling mechanism has become a central tenet in G-protein research, and GPCRs have become major pharmaceutical targets for drug discovery [4].

The eukaryotic unicellular yeast, *Saccharomyces cerevisiae*, also shares the G-protein-mediated signal transmission mechanism with higher mammalian cells [3]. It is notable that *S. cerevisiae* offers a crucial advantage to simplify the study of GPCR signaling because it expresses only one kind of G-protein, which thereby avoids potential problems such as signaling cross-talk in mammalian cells [5]–[8]. Therefore, *S. cerevisiae* is a suitable host cell for the screening of functional residues in GPCRs [5], [9], [10].

Yeast cell-surface display technology is a powerful platform that enables proteins expressed in yeast to be tethered onto the cell surface [11]–[15]. This is accomplished by the use of “anchor” proteins that naturally localize on the cell surface in yeast cells. Typically, the gene encoding the target protein is fused to the anchor protein together with a secretion signal sequence at the N-terminus to both enable secretion of the fusion protein and to tether it firmly to the cell surface. As typical anchor proteins, the C-terminal domains of truncated α-agglutinin (Sag1p; a mannoprotein involved in sexual adhesion) and truncated Flo1p (a lectin-like cell-wall protein involved in flocculation) containing the glycosyl-phosphatidylinositol (GPI) anchor attachment signal sequence at the C-terminus are fused to the target protein at their N-termini [16], [17]. Regarding other anchor proteins, the Flo1p flocculation functional domain without the GPI anchor attachment signal (FS anchor) permits the fusion of the target protein to both its N- and C-termini [18]. These anchor proteins are used to display the target proteins on the yeast cell wall. In contrast, periplasmic invertase (Suc2 anchor) can be fused to both the N- and C-termini of a target protein, enabling it to localize into the periplasmic space [19]. To date, yeast cell-surface display technology has been adopted for a broad range of applications including enzymatic catalysis, immune adsorption and protein engineering [16]–[18], [20]–[23].

**Figure 1:**
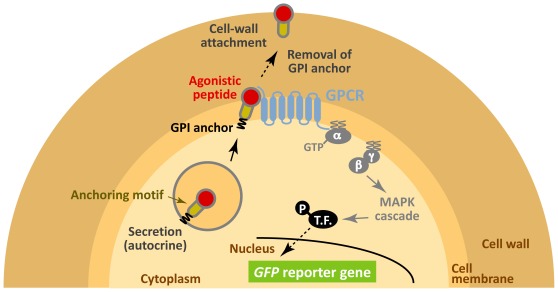
Schematic illustration of our concept using yeast cell-surface display technology to selectively track eligible agonistic peptides for human GPCRs by assembling the autonomous signaling complex within individual cells (cell wall trapping of autocrine peptides (CWTrAP) strategy). The candidate autocrine peptides fused with the anchoring proteins are processed via secretion pathways in engineered yeast cells. Their agonistic activities for heterologously-expressed human GPCRs are discerned on yeast cell membranes. Only when the peptide possesses objective agonistic activity, membrane-peripheral G-proteins promote intracellular signaling and induce transcription of the GFP reporter gene. Because the autocrine peptides are automatically trapped onto individual yeast cell walls, the captured peptides are unable to diffuse toward surrounding yeast cells that express the target human GPCR and any other peptides. T.F. indicates transcription factor.

Here, we describe a unique concept using yeast cell-surface display technology to selectively track eligible peptides that present agonistic activity for human GPCRs. In our system, individual yeast cells expressing human GPCRs fulfill a series of roles from the manufacture of peptides to the sensing of agonistic activity. Briefly, yeast cells synthesize candidate peptides in fusion with a secretion signal sequence and an anchoring motif. Agonistic peptides are capable of binding cell surface GPCRs that transduce the signal into the cell. Finally, the yeast traps the signaling peptide on its cell wall (Figure 1). Here, we use a yeast strain that is engineered to express a green fluorescent protein (*GFP*) reporter gene in response to GPCR activation. Therefore, stimulation by agonistic peptides can be recognized by the generation of a green fluorescence signal [3]. In principle, because signaling peptides are unable to diffuse to surrounding cells, our strategy has the potential to build autonomous signaling complexes on a cell-by-cell basis. Our peptide trapping method (cell wall trapping of autocrine peptides (CWTrAP) system) will allow the identification of lead peptides from combinatorial peptide libraries as starting points for drug screening.

## Results and Discussion

**Figure 2:**
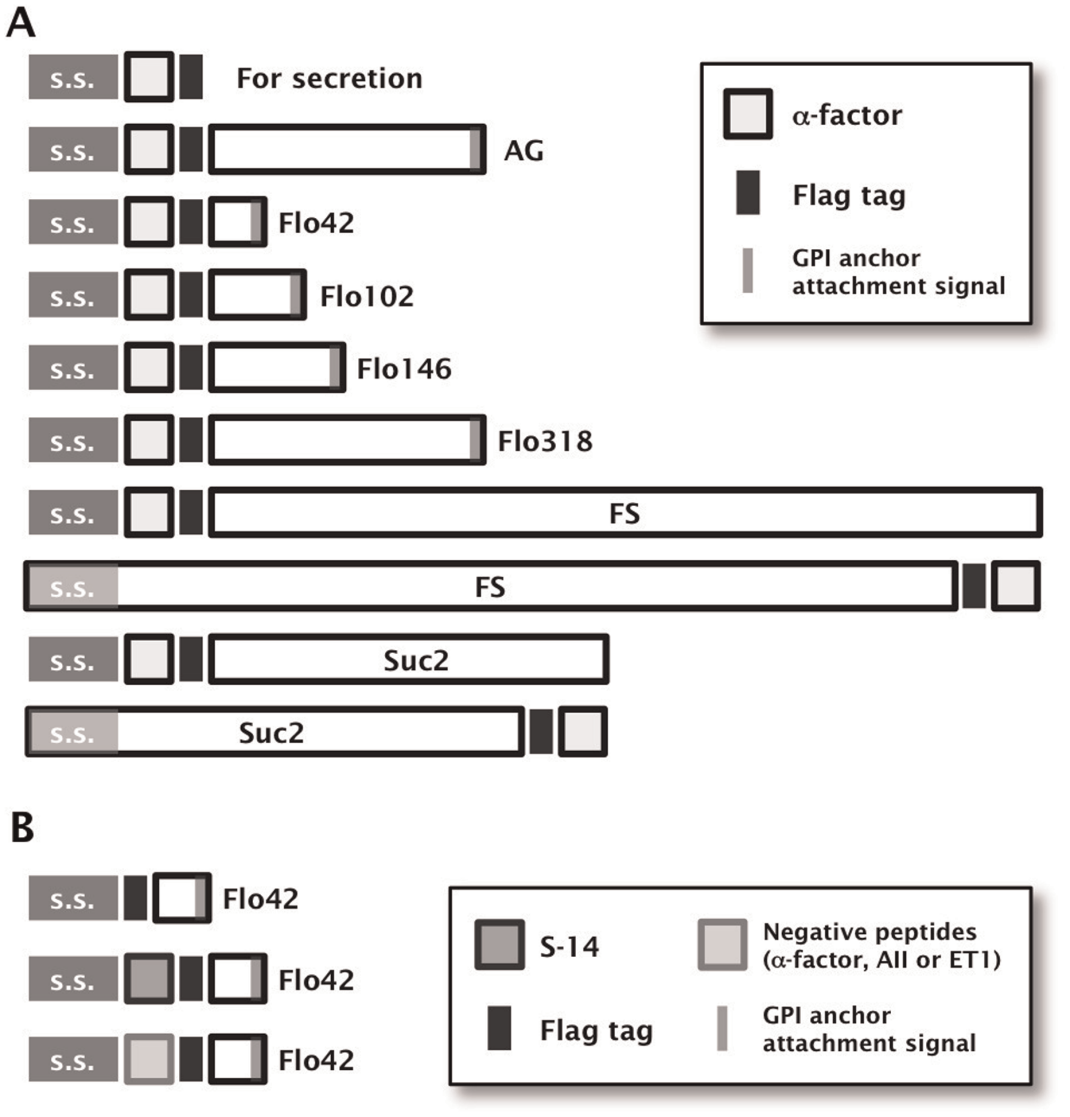
Schematic illustration of the fusion protein constructs used to display agonistic peptides on the yeast cell-surface. (A) Constructs for displaying α-factor peptides. AG: C-terminal half of α-agglutinin anchor. s.s.: secretion signal sequence. The pre-pro-region derived from α-factor was used as s.s. For the fusion of FS and Suc2 anchors to the α-factor peptides at their C-termini, the original s.s. encoded in the N-termini of Flo1p or Suc2p were used, respectively. The uppermost construct for secretion of α-factor peptide contains no anchoring motifs. All constructs contain the Flag tag. (B) Constructs for displaying S-14 by the Flo42 anchor. The upper construct displaying only Flag and Flo42 peptides was used as a negative control for the SSTR5 signaling assay. The middle and lower constructs displaying, respectively, eligible peptide (S-14) and negative control peptides (α-factor, AII and ET1) by Flag–Flo42 fusion proteins were also used for the SSTR5 signaling assay.

To corroborate the viability of cell-surface display technology to track agonistic activity for GPCRs (CWTrAP system), we used α-factor pheromone, a natural ligand for the endogenous yeast 7-transmembrane GPCR, Ste2, which is specifically expressed in the **a**-type-strain [24]. In nature, α-type yeast strains secrete α-factor to induce mating signal transduction in the **a**-type strain by binding to the Ste2 receptor on its cell surface [25]. The ability of several types of protein motifs to anchor and transduce the autocrine α-factor were tested in the recombinant **a**-type yeast cells, which can express a *GFP* reporter gene in response to pheromone signaling (Figure 1). All constructs of fusion proteins that displayed α-factor peptides were designed to contain a Flag tag between the α-factor peptides and anchor proteins (Figure 2A and Table 1).

**Table 1:** Yeast strains and plasmids used in this study.

Strain or plasmid	Relative feature	Source
**Yeast strain**		
BY4741	*MAT* **a** *his3*Δ*1 leu2*Δ*0 met15*Δ*0 ura3*Δ*0*	[31]
IMG-4	BY4741 *fus1*::*FUS1-EGFP-T_GAPDH_-HIS3 bar1*Δ::*LEU2 far1*Δ::*kanMX4*	This study
IMG-50	BY4741 *fus1*::*FUS1-EGFP-T_GAPDH_-HIS3 sst2*Δ::*AUR1-C ste2*Δ::*LEU2*	[28]
IMFD-70	BY4741 *fig1*Δ::*EGFP his3*Δ::*P_FIG1_-EGFP far1*Δ *sst2*Δ::*AUR1-C ste2*Δ::*LEU2*	[5]
**Plasmid**		
pESC-URA^a^	Expression vector containing *GAL1-GAL10* divergent promoter, 2µ origin and *URA3* marker	Agilent Technologies
pUESCαsf	pESC-URA, α-factor–Flag peptide expression (for secretion)	This study
pUESCαf-AG	pESC-URA, α-factor–Flag–AG^b^ fusion protein expression (for display)	This study
pUESCαf-FLO42	pESC-URA, α-factor–Flag–Flo42 fusion protein expression (for display)	This study
pUESCαf-FLO102	pESC-URA, α-factor–Flag–Flo102 fusion protein expression (for display)	This study
pUESCαf-FLO146	pESC-URA, α-factor–Flag–Flo146 fusion protein expression (for display)	This study
pUESCαf-FLO318	pESC-URA, α-factor–Flag–Flo318 fusion protein expression (for display)	This study
pGK421^a^	Expression vector containing *PGK1* promoter, 2µ origin and *MET15* marker	[5], [6]
pGK-SSTR5-HA	pGK421, SSTR5-HA human receptor expression	[5], [6]
pGK426^a^	Expression vector containing *PGK1* promoter, 2µ origin and *URA3* marker	[36]
pGK42	pGK426, Flag–Flo42 anchor protein expression (for display)	This study
pGK-S1442	pGK426, S-14–Flag–Flo42^c^ fusion protein expression (for display)	This study
pGK-alpha42	pGK426, α-factor–Flag–Flo42 fusion protein expression (for display)	This study
pGK-AII42	pGK426, AII–Flag–Flo42^d^ fusion protein expression (for display)	This study
pGK-ET142	pGK426, ET1–Flag–Flo42^e^ fusion protein expression (for display)	This study
pMHG-FIG1	Multi-copy reporter plasmid containing *FIG1* promoter, *GFP* reporter gene, 2µ origin and *HIS3* marker	[6]

All transcription products for display or secretion contain the secretion signal sequence of α-factor.

a The indicated vectors were used as mock controls.

b AG indicates C-terminal half of α-agglutinin anchor protein.

c S-14 encodes somatostatin 14 mature peptide.

d AII encodes angiotensin II mature peptide.

e ET1 encodes endothelin-1 mature peptide.

**Figure 3:**
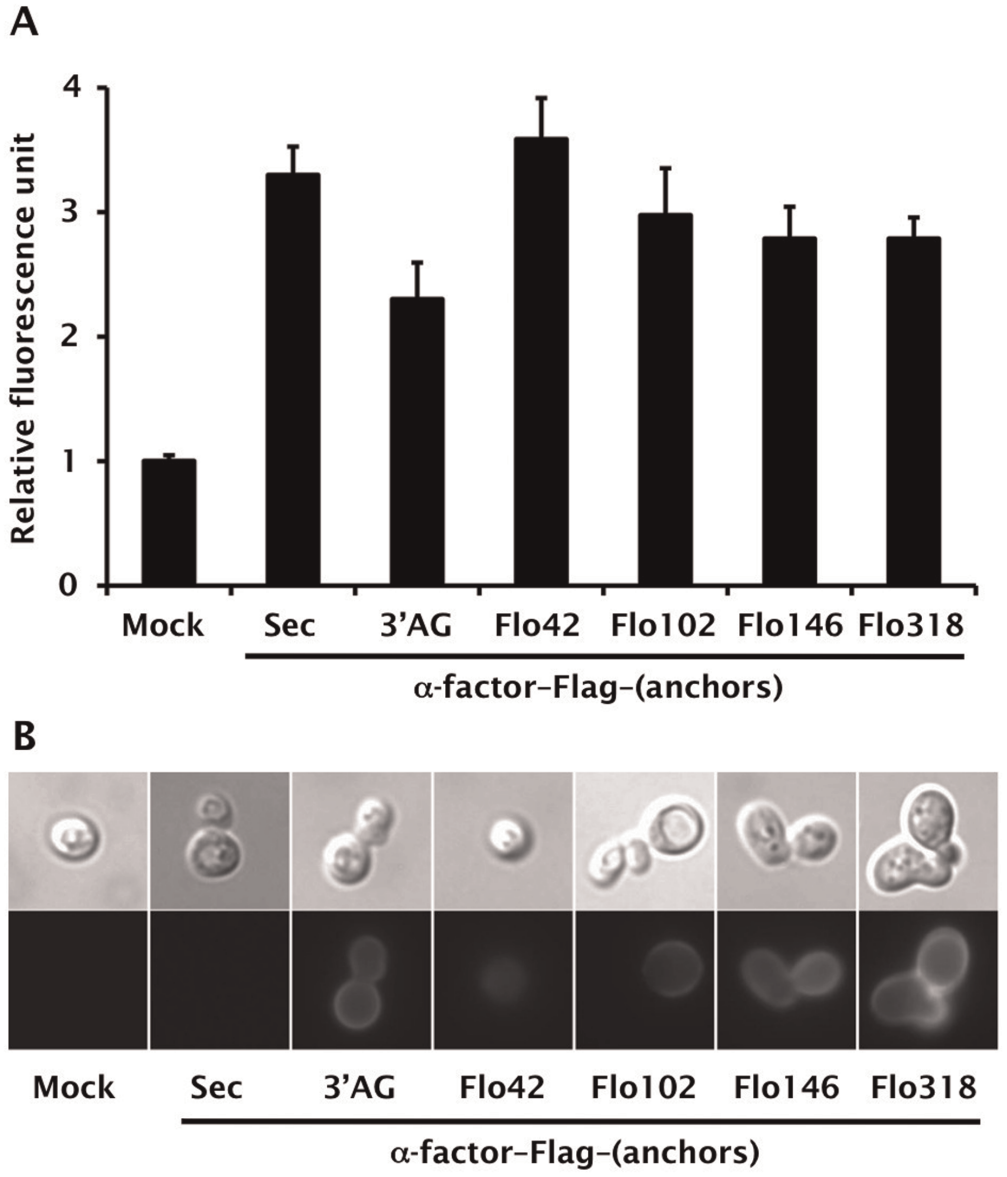
Evaluation of the CWTrAP system using α-factor peptide for yeast endogenous Ste2 receptor. (A) Pheromone signaling assays of α-factor-displaying yeast strains. Error bars represent the standard deviation of three independent experiments. (B) Immunofluorescence staining of α-factor displaying yeast strains. Anti-Flag antibody and Alexa Fluor 546-conjugated secondary antibody were used for detection of secreted α-factor or α-factor-anchor fusion proteins. IMG-4 was used as the host strain. The transformants used in these experiments are listed in Table S3. Sec: free, secreted form of α-factor. AG: C-terminal half of α-agglutinin anchor.

We used the IMG-4 yeast strain to display α-factor pheromone on its cell surface because this strain can monitor signaling levels through its endogenous Ste2 receptor via a *GFP* reporter gene (Table 1). To test our concept, we evaluated the C-terminal 320 aa of Sag1p (C-terminal half of α-agglutinin; AG) [16] and various lengths of truncated Flo1p derivatives (C-terminal 42, 102, 146 and 318 aa of Flo1p; Flo42, Flo102, Flo146 and Flo318) [17] as anchor proteins with GPI anchoring motifs (Figure 2A and Table 1). A recombinant yeast strain, engineered to express the α-factor autocrine peptide with a secretion signal sequence but lacking an anchor motif, robustly generated a higher green fluorescence signal than a strain harboring a mock plasmid (Figure 3A, Mock and Sec). Immunofluorescence staining of Flag-tagged α-factor peptide revealed no fluorescence on the surface of engineered yeast cells (Figure 3B, Sec). These results suggest that secreted α-factor could bind the endogenous Ste2 receptor and transduce the signal inside the yeast cells.

Next, we tested the cell wall trapping (CWTrAP) strategy for α-factor peptide with GPI anchoring motifs. All engineered yeast strains expressing α-factor peptides fused to the N-termini of the anchor proteins (AG and Flo42–318) with an inserted Flag tag (Figure 2A) successfully generated a green fluorescence signal (Figure 3A), confirming that the fusion peptide is able to activate signal transduction in yeast. Using GFP fluorescence intensity as an indicator of signaling strength, shorter anchor peptides appeared more capable of activating the GPCR (Figure 3A). The α-factor peptide fused to Flag and Flo42 exhibited higher responsiveness compared to α-factor lacking the anchor protein. This interesting result may arise from the transient enrichment of the GPI-anchored peptide on the yeast cell membrane, although the GPI-anchored peptide should be cleaved from the plasma membrane by phosphatidylinositol-specific phospholipase C (PI-PLC) and tethered on the cell wall [11]–[13].

Although shorter peptides tend to make detection of the Flag tag more difficult, due to the report that shorter peptides can bury the tag within the cell wall [17], we were able to confirm an anchor-dependent association with the yeast cell wall by immunofluorescence staining (Figure 3B). Because peptides anchored to the cell wall are unable to diffuse to surrounding cells, this result emphasizes the viability of our concept for the assembly of the autonomous signaling complex within individual yeast cells. Additionally, we verified that a subset of Flo42 was highly glycosylated (Figure S1); however, the agonistic activity of the α-factor peptide was unlikely to be affected by the posttranslational glycosylation of the anchor protein.

Next, we tested additional motifs that permit peptides to be fused to both the N- and C-termini of the anchor proteins. We replaced the GPI anchor proteins with the FS anchor [18] and the Suc2 anchor [19] (Figure 2A, Table S2 and Document S1). Signal transduction was more efficient when using the FS anchor, compared to the Suc2 anchor (Figure S2). These results show that agonistic peptides can be fused to both the N- and C-termini of anchor proteins. Even though the FS anchor (1099 aa) served as an efficient motif for transducing α-factor peptide signaling, we used the Flo42 anchor motif, whose molecular mass is much lower (Figure 2A), in all following experiments in order to minimize the possibility of steric hindrance.

To further demonstrate the viability of our concept, the IMFD-70 yeast strain, which can monitor signaling levels from recombinantly expressed heterologous GPCRs by a *GFP* reporter gene [5] (Table 1), was used to test if signal transmission from human GPCRs expressed on the yeast cell surface was possible. For these experiments, human somatostatin receptor subtype 5 (SSTR5), and the natural intramolecular-cross-linked cyclic peptide ligand, somatostatin 14 (S-14), were used [26], [27].

**Figure 4:**
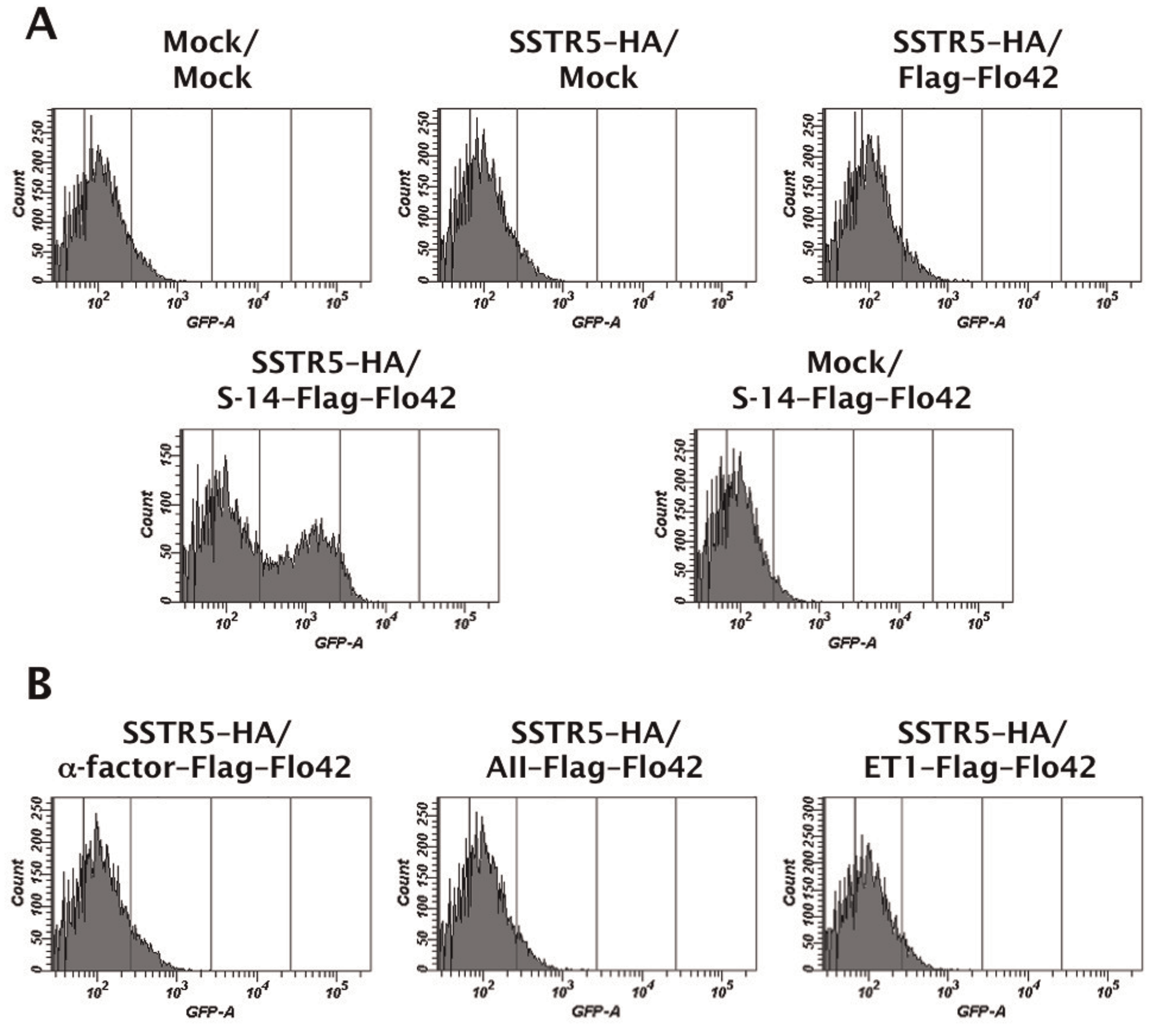
Evaluation of the CWTrAP system using somatostatin peptide for the human SSTR5 receptor. (A) SSTR5 signaling assays of the cyclic somatostatin peptide displaying yeast strain and control strains. (B) SSTR5 signaling assays of non-target peptide displaying yeast strains. IMFD-70 was used as the host strain. The transformants used in these experiments are listed in Table S3. S-14 indicates 14 aa of somatostatin cyclic peptide, α-factor indicates 13 aa of yeast pheromone peptide, AII indicates 8 aa of angiotensin II peptide, and ET1 indicates 21 aa of endothelin-1 peptide.

To express the autocrine somatostatin and trap it on the yeast cell wall, we designed the S-14 peptide with an N-terminal secretion signal sequence and a C-terminal Flo42 anchor protein with a Flag tag (Figure 2B and Table 1). We constructed several negative controls by eliminating the S-14 peptide or by replacing it with agonistic peptides for other GPCRs (Figure 2B and Table 1). We expressed hemagglutinin (HA)-tagged human SSTR5 on the yeast cell surface using previously reported plasmids [5], [6] (Table 1). We used these expression and mock plasmids to investigate the ability of the S-14–Flag–Flo42 autocrine peptide to activate GPCR signaling (Figure 4).

As shown in Figure 4A, the engineered yeast strain concomitantly expressing SSTR5–HA and S-14–Flag–Flo42 successfully induced *GFP* reporter gene expression, whereas the other control strains possessing either SSTR5–HA or S-14–Flag–Flo42 did not. Similarly, a control strain expressing SSTR5–HA and the autocrine Flag–Flo42 fusion protein lacking the S-14 peptide was unable to express a green fluorescence signal (Figure 4A). These results demonstrate that autocrine activation of recombinant SSTR5 by binding of the S-14 peptide fused to the Flo42 anchor mediates pheromone signaling via endogenous peripheral G-proteins in yeast [5]. Furthermore, we were able to confirm the specificity of the S-14 peptide because three control peptides in which the S-14 peptide was replaced with the yeast Ste2 receptor agonist, α-factor, the human angiotensin receptor agonist, angiotensin II (AII), or the human endothelin receptor agonist, endothelin-1 (ET1), did not generate a green fluorescence signal (Figure 4B).

**Figure 5:**
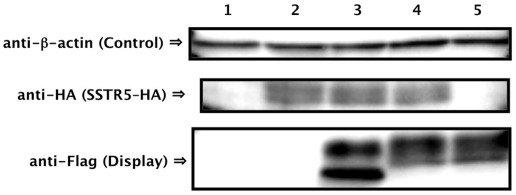
Confirmation of protein expression. Western blots of extracts from somatostatin displaying yeast strains. Lane 1: Mock/Mock, 2: SSTR5/Mock, 3: SSTR5/Flag–Flo42, 4: SSTR5/S-14–Flag–Flo42, 5: Mock/S-14–Flag–Flo42. Anti-β-actin antibody was used as loading control. Anti-HA antibody was used for detection of SSTR5 receptor. Anti-Flag antibody was used for detection of Flag–Flo42 anchor or S-14–Flag–Flo42 fusion proteins. IMFD-70 was used as the host strain. The transformants used in these experiments are listed in Table S3.

**Figure 6:**
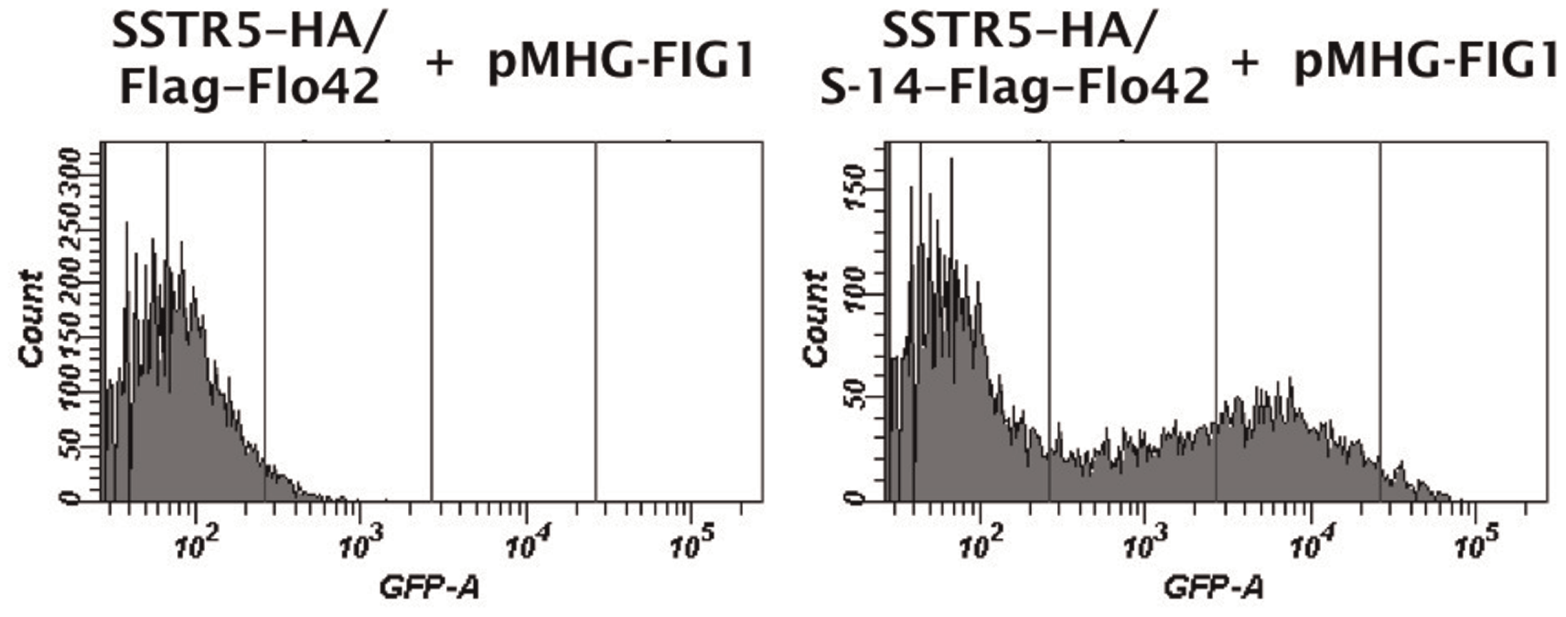
Improved fluorescence signal in the CWTrAP system using somatostatin peptide for the human SSTR5 receptor. SSTR5 signaling assays of the cyclic somatostatin peptide displaying yeast strain and the non-displaying control strain, which contain the multi-copy plasmid harboring a *GFP* reporter gene cassette (pMHG-FIG1). IMFD-70 was used as the host strain. The transformants used in these experiments are listed in Table S3.

We confirmed the expression of SSTR5–HA receptor and S-14–Flag–Flo42 fusion protein by western blot analysis (Figure 5). Equal loading of the sodium lauryl sulfate (SDS)-extracted cell lysate fraction from each pellet was confirmed using anti-β-actin. SSTR5–HA receptor (anti-HA; lanes 2–4) and Flag–Flo42 anchor or S-14–Flag–Flo42 fusion proteins (anti-Flag; lanes 3–5) were successfully detected in the extracts of each appropriate transformant. The two unequal bands detected by the anti-Flag antibody in the Flag–Flo42 and S-14–Flag–Flo42 transformants likely represent the signal-cleaved and -uncleaved proteins, because the pre-pro-region derived from α-factor was used as the secretion signal sequence. We therefore tested the ability of the other active somatostatin isoform S-28 [26] and other secretion signal sequences (pre-region of α-factor and signal sequences derived from *S. cerevisiae* Suc2p and *Rhizopus oryzae* glucoamylase) to mediate signal transduction in the IMG-50 yeast strain. This strain has a slightly different genetic background to IMFD-70 (*FAR1*-intact strain [28], the description of the *far1*Δ allele can be found in Materials and Methods; Table 1), but the expression profiles of the *GFP* reporter genes remained essentially unchanged (Figure S3). Also, the insertion of GS linkers (GGGGS and GGGSGGGGS) between the S-14 peptide and Flag–Flo42 did not improve *GFP* expression (Figure S4). Because GPCR signaling has been reported to decrease plasmid retention even in the *far1*Δ yeast strain [28], false-negative signals (non-signaling cell cluster; Figure 4A, SSTR5–HA/S-14–Flag–Flo42) may be caused by plasmid loss. Because other secretion signal sequences and the insertion of GS linkers had no effect on expression of the *GFP* reporter gene, it is unlikely that a false-negative signal would be caused by steric hindrance of the S-14 peptide (Figure S3 and S4). Nevertheless, the presence of false-negative cells within an identical cell cluster implies that peptides captured on the cell wall have little influence on the surrounding cells (Figure 4, S3 and S4). Therefore, we demonstrated that peptides captured on the cell wall did not induce false-positive signals in surrounding non-target cells, even when two types of cells, one expressing the S-14–Flag–Flo42 (target cells) and the other expressing the Flag–Flo42 anchor lacking S-14 (non-target cells or surrounding cells), were mixed (Figure S5). Additionally, we successfully enhanced the weaker green fluorescence signal of the IMFD-70 strain expressing SSTR5–HA and S-14–Flag–Flo42 (Figure 4A) by concurrently introducing a multi-copy plasmid harboring the *GFP* reporter gene cassette (pMHG-FIG1 [6]) (Figure 6). These results strongly support the feasibility of our conceptual CWTrAP system to identify eligible agonistic peptides for human GPCRs.

**Figure 7:**
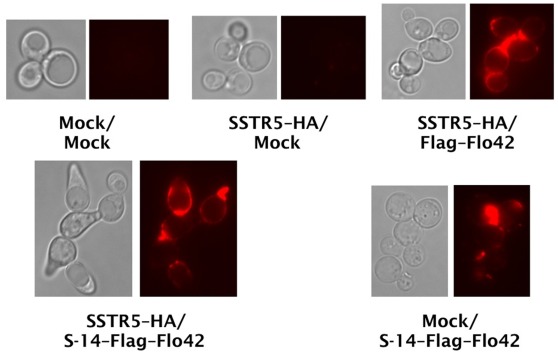
Confirmation of peptide trapping on yeast cell surfaces. Immunofluorescence staining of somatostatin displaying yeast strains. Anti-Flag antibody and Alexa Fluor 594-conjugating secondary antibody were used for detection of Flag–Flo42 anchor or S-14–Flag–Flo42 fusion proteins. Red fluorescence images are shown in false-color. IMFD-70 was used as the host strain. The transformants used in these experiments are listed in Table S3.

Finally, to examine whether the yeast cell wall did indeed trap the autocrine peptide fused to the Flo42 anchor, transformants were analyzed by immunofluorescence staining with anti-Flag primary antibody and Alexa Fluor 594 conjugated secondary antibody (Figure 7). We observed red fluorescence on the cell surfaces of appropriate transformants that expressed Flag–Flo42 anchor or S-14–Flag–Flo42 fusion proteins. In addition, we only observed a morphology change [29] on cells expressing both SSTR5–HA and S-14–Flag–Flo42, supporting our hypothesis that the autocrine S-14 peptide specifically triggered signal transduction via the SSTR5 receptor in the recombinant yeast cells. Thus, we successfully verified that the S-14 autocrine peptide fused to the Flo42 anchor protein was trapped on the yeast cell wall.

In this study, we have demonstrated how a strategy for cell wall trapping of autocrine peptides (CWTrAP system) functions to discern agonistic activity for human GPCRs expressed in yeast cells, by using the intramolecular-cross-linked cyclic peptide S-14 and its specific receptor as our model. Our motivation was to selectively track eligible agonistic peptides for human GPCRs by assembling an autonomous signaling complex within individual cells. By combining cell-surface display technology and established yeast combinatorial genetic engineering technology with flow cytometric single-cell screening [30], we aim to identify eligible peptides from peptide libraries. Here, the feasibility of our concept is demonstrated by peptide capture, and subsequent signal transduction, by heterologously-expressed human GPCRs, which prevent the captured peptides from diffusing to surrounding yeast cells and eliciting a false-positive response. Therefore, the captured peptides are successfully presented by yeast cell-surface display technology.

## Materials and Methods

### Media

Synthetic raffinose (SR) media contained 6.7 g/l yeast nitrogen base without amino acids (YNB) (BD-Diagnostic Systems, Sparks, MD, USA) and 20 g/l raffinose. For SRGC media, 20 g/l galactose and 20 g/l casamino acids (BD-Diagnostic Systems) were added into SR media. Synthetic dextrose (SD) media contained 6.7 g/l YNB and 20 g/l glucose. For SDM71 media, SD media was adjusted to pH 7.1 with 200 mM MOPSO buffer (Nacalai Tesque, Kyoto, Japan). Amino acids and nucleotides (20 mg/l histidine, 60 mg/l leucine, 20 mg/l methionine or 20 mg/l uracil) were supplemented into each medium to provide the relevant auxotrophic components.

### Yeast Strains

Yeast strains used for assays were generated from BY4741 [31] as a parental backbone strain and are listed in Table 1. The transformation procedure using linear DNA fragments followed the lithium acetate method [32]. All primers used for the strain constructions are listed in Table S1. The *bar1*Δ alleles that relieve the degradation of α-factor pheromone [33] were conferred to BY4741*far1*Δ (obtained from *Saccharomyces* Genome Deletion Project [34]) by homologous recombination with the amplified *LEU2* fragments, producing the IM-4 strain. The *FUS1-GFP* reporter gene was integrated into the *FUS1* genomic loci of IM-4 with a fragment prepared by digestion of pUC119-FUS1-EGFP-HIS3 [28] with EcoRI and SphI, producing the IMG-4 strain. The *P_FUS1_-FUS1-GFP* or *P_FIG1_-GFP* reporter gene was used to monitor signal transduction promoted by stimulating GPCRs in yeast (IMG-4, IMG-50 or IMFD-70 [5]). *far1*Δ alleles were used to avoid G1 arrest and promote cell-cycle progression during signal activation [5], [28], [35] (IMG-4 and IMFD-70). *sst2*Δ and *ste2*Δ alleles were used to obtain hypersensitivity for ligand stimulation and to inhibit competitive expression of endogenous yeast GPCRs [5], [28] (IMG-50 and IMFD-70).

### Plasmids

All plasmids used for assays are listed in Table 1. All primers used for plasmid constructions are listed in Table S1. The amplified pre, pro (containing secretion signal sequence, s.s.) and first mature sequences of α-factor peptide including a C-terminal Flag tag and stop codon were inserted into the pESC-URA yeast expression vector (Agilent Technologies, Santa Clara, CA, USA) at the BamHI and XhoI sites, creating pUESCαsf. As the backbone for α-factor-displaying plasmids, pUESCαf and pUESCαf(AG) without stop codons were constructed in essentially the same manner. The amplified genes encoding Flo42, Flo102, Flo146 and Flo318 anchors were inserted into pUESCαf at the XhoI and NheI sites, resulting in pUESCαf-FLO42, -FLO102, -FLO146 and -FLO318, respectively. pUESCαf-AG was produced in a similar procedure by inserting the gene encoding the C-terminal 320 aa of Sag1p (C-terminal half of α-agglutinin anchor, AG) into pUESCαf(AG) at the XhoI and NheI sites. As the backbone for somatostatin-displaying plasmids, we constructed pGK426-tgFLO42 by inserting the amplified *FLO42* anchor gene with *FLAG* at the N-terminus into pGK426 at the SalI and BglII sites [36]. The DNA fragment containing s.s. of α-factor and S-14 mature peptide was amplified by overlapping PCR and inserted into pGK426-tgFLO42 at the NheI and SalI sites, producing pGK-S1442. We generated pGK-alpha42 as an α-factor peptide-displaying control plasmid, using essentially the same procedure. As other peptide-displaying control plasmids, the gene containing s.s. of α-factor and the mature peptide sequences of angiotensin II (AII) or endothelin-1 (ET1) was inserted into pGK426-tgFLO42 at the NheI and SalI sites, generating pGK-AII42 and pGK-ET142, respectively. As a peptide-non-displaying control plasmid, pGK42 was created in a similar procedure by using the DNA fragment containing s.s. of α-factor without the peptide sequence. pGK-SSTR5-HA [5], [6] was used to express human SSTR5 receptor fused to a C-terminal HA tag. Transformation of plasmids was performed using the lithium acetate method. All transformants used for assays are listed in Table S3.

### Pheromone Signaling Assay

To assay signal activation from the endogenous Ste2 pheromone receptor, the IMG-4 yeast strains harboring the pESC-URA-based plasmids were grown in SR media at 30°C, and cells were then inoculated into 100 ml of SRGC media to give an initial optical density of 0.03 at 600 nm. Cultures were grown at 30°C with shaking at 150 opm for 72 h. The cells were collected and diluted into test tubes containing sheath solution and GFP fluorescence was measured using a BD FACSCalibur flow cytometer (BD Biosciences, San Jose, CA, USA). The green fluorescence signal from 10,000 cells was excited with an argon laser and collected through a 530/30 nm band-pass (FL1) filter. The data were analyzed using BD CELLQuest software (BD Biosciences). The “relative fluorescence unit” was defined using the FL1-H geometric mean of IMG-4 harboring mock plasmid (pESC-URA) as the benchmark.

### SSTR5 Signaling Assay

To assay signal activation from human SSTR5 receptor, the IMFD-70 yeast strains harboring the pGK-SSTR5-HA and pGK426-based plasmids were grown in SD media at 30°C, and cells were then inoculated into 20 ml of SDM71 media to give an initial OD_600_ of 0.03. Cultures were grown at 30°C with shaking at 150 opm for 15 h. The cells were collected and diluted into test tubes containing sheath solution and GFP fluorescence was measured using a BD FACSCanto II flow cytometer (BD Biosciences). The green fluorescence signal from 10,000 cells was excited with a blue laser and collected through a 530/30 nm band-pass (GFP) filter. The data were analyzed using BD FACSDiva software (BD Biosciences).

### Western Blotting

Collected cells were suspended in 10 mM Tris-HCl (pH 7.8) containing 1 mM phenylmethylsulfonyl fluoride (PMSF) to give an OD_600_ of 5, and 200 µl of cell suspension was disrupted using a Multi-beads shocker (Yasui Kikai, Osaka, Japan) with 0.5 mm glass beads. Cell lysates were centrifuged at 1,000×g for 5 min and the pellet was then washed three times with 10 mM Tris-HCl containing 1 mM PMSF. The pellet was resuspended in 200 µl of SDS solubilization buffer (50 mM Tris-HCl [pH 7.8], 2% SDS [w/v], 100 mM ethylene diamine tetraacetic acid [EDTA], 40 mM 2-mercaptoethanol [2-ME]), and the suspension was boiled at 95°C for 5 min and then centrifuged at 10,000×g for 5 min. The supernatant was collected and diluted with an equivalent volume of 2× sample buffer (25 mM Tris-HCl [pH 6.8], 4% SDS [w/v], 20% glycerol [w/v], 10% 2-ME [v/v], 0.1 mg/ml bromophenol blue [BPB]). Twenty microliters of each sample was loaded onto a 12.5% SDS-polyacrylamide gel and proteins were separated by electrophoresis and then transferred to polyvinylidene fluoride (PVDF) membrane (Immobilon-FL; Millipore, Billerica, MA, USA) by electroblotting. Western blots were performed as follows: mouse anti-β-actin monoclonal antibody (Abcam, Cambridge, UK) as loading control, rabbit anti-HA antibody (Bethyl Laboratories, Montgomery, TX, USA) for HA-tagged SSTR5 receptor, and mouse anti-Flag M2 monoclonal antibody (Sigma-Aldrich, St. Louis, MO, USA) for fusion proteins with S-14 peptide, Flag tag and Flo42 anchors were primarily used at dilutions of 1∶5,000 in TBST (10 mM Tris-HCl [pH 8.0], 150 mM NaCl, 0.05% Tween-20 [v/v]). Anti-mouse or anti-rabbit secondary antibodies conjugated with alkaline phosphatase (Promega, Madison, WI, USA) were used at dilutions of 1∶5,000 in TBST. Chemiluminescent visualization was performed with Amersham CDP-Star Detection Reagent (GE Healthcare, Buckinghamshire, UK) and the signal was detected using a lumino-image analyzer LAS-1000mini system (Fujifilm, Tokyo, Japan).

### Immunofluorescent Staining

For α-factor displaying yeasts (IMG-4), collected cells were diluted to give an OD_600_ = 10 with distilled water and the cell suspension was used for immunofluorescence staining by incubating with mouse anti-Flag M2 monoclonal antibody (Sigma-Aldrich) at a dilution of 1∶500 for 1 h at room temperature. After washing in triplicate, anti-mouse secondary antibody conjugated with Alexa Fluor 546 (Invitrogen Life Technologies, Carlsbad, CA, USA) at a dilution of 1∶500 was incubated with the cell suspensions for 1 h at room temperature. After washing in triplicate, cells were resuspended in distilled water and observed on a fluorescence microscope with a monochrome CCD camera. To obtain micrographs of better clarity, essentially the same procedure was used for somatostatin displaying yeasts (IMFD-70), but the density of the collected cells was adjusted to OD_600_ = 5. Antibodies were used at a dilution factor of 1∶100. Anti-mouse IgG conjugated with Alexa Fluor 594 (Invitrogen Life Technologies) was used as the secondary antibody.

## Supporting Information

Figure S1 **Western blotting of SDS-extracted fractions from the IMG-4/pUESCαf-FLO42 yeast strain.** EndoH_f_ (Endoglycosidase H) was used to confirm glycosylation of the Flo42 anchor. Anti-Flag M2 monoclonal antibody and anti-mouse secondary antibody conjugated with alkaline phosphatase were used to detect the α-factor–Flag–Flo42 fusion protein. NBT (nitro blue tetrazolium) and BCIP (5-bromo-4-chloro-3-indolyl-phosphate) were used for the colorimetric reaction.
(TIF)

Figure S2 **Pheromone signaling assays of α-factor-displaying yeast strains with various anchor motifs (color histograms).** Gray histograms show the data from control strains (mock). IMG-4 was used as the host strain. The transformants used in this experiment are listed in Table S3.
(TIF)

Figure S3 **SSTR5 signaling assays of somatostatin-displaying yeast strains with various secretion signal sequences (color histograms).** The Flo42 anchor was used for somatostatin display. S-28 indicates the 28 aa active isoform of somatostatin peptide. Gray histograms show the data from control strains (mock). Cultures were grown in SDM71 media for 22 h. IMG-50 was used as the host strain. The transformants used in this experiment are listed in Table S3.
(TIF)

Figure S4 **SSTR5 signaling assays of somatostatin-displaying yeast strains with different length GS linkers (color histograms).** The S-14 peptide and Flo42 anchor were used for display. Gray histograms show the data from control strains (mock). Cultures were grown in SDM71 media for 12 h. IMG-50 was used as the host strain. The transformants used in this experiment are listed in Table S3.
(TIF)

Figure S5 **SSTR5 signaling assays of somatostatin-displaying yeast strain (target cells) mixed with somatostatin-non-displaying strain (non-target cells).** S-14–Flag–Flo42 and Flag–Flo42 fusion proteins were used as target and non-target cells, respectively. R1 regions in the dot plots show the gates for FACS sorting. The ratio of initial cell densities was adjusted to 10∶1 (non-target cells : target cells), and the cultures were grown in SDM71 media. IMG-50 was used as the host strain. The transformants used in this experiment are listed in Table S3.
(TIF)

Table S1 **List of primers.**
(PDF)

Table S2 **Plasmids used in Supplementary data.**
(PDF)

Table S3 **List of strains and transformants used for assays.**
(PDF)

Document S1 **Supplementary Materials and Methods (Plasmid constructions for supporting information).**
(PDF)
