# Supplementary figures and images for: Cell Wall Trapping of Autocrine Peptides for Human G-Protein-Coupled Receptors on the Yeast Cell Surface
Source: PLoS One. 2012 May 18;7(5):e37136. doi: 10.1371/journal.pone.0037136 (PMC3356411; doi:10.1371/journal.pone.0037136)

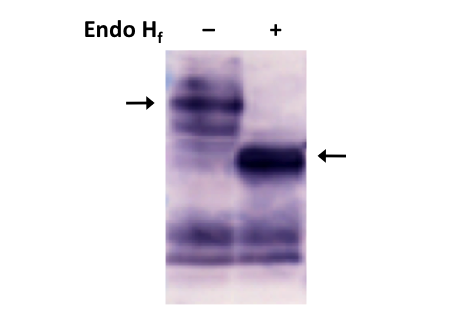

Supplement: Figure S1 — Western blotting of SDS-extracted fractions from the IMG-4/pUESCαf-FLO42 yeast strain. EndoHf (Endoglycosidase H) was used to confirm glycosylation of the Flo42 anchor. Anti-Flag M2 monoclonal antibody and anti-mouse secondary antibody conjugated with alkaline phosphatase were used to detect the α-factor–Flag–Flo42 fusion protein. NBT (nitro blue tetrazolium) and BCIP (5-bromo-4-chloro-3-indolyl-phosphate) were used for the colorimetric reaction. (TIF) [file pone.0037136.s001.tif]

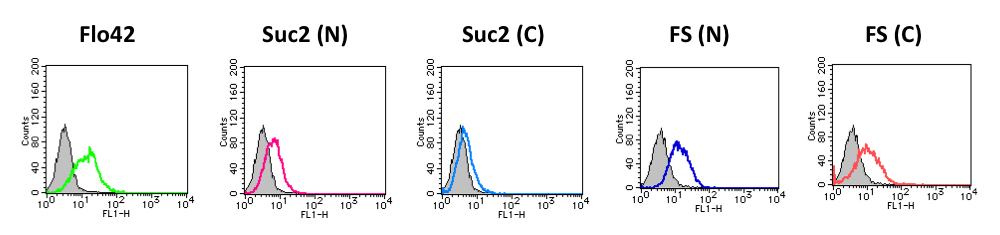

Supplement: Figure S2 — Pheromone signaling assays of α-factor-displaying yeast strains with various anchor motifs (color histograms). Gray histograms show the data from control strains (mock). IMG-4 was used as the host strain. The transformants used in this experiment are listed in Table S3. (TIF) [file pone.0037136.s002.tif]

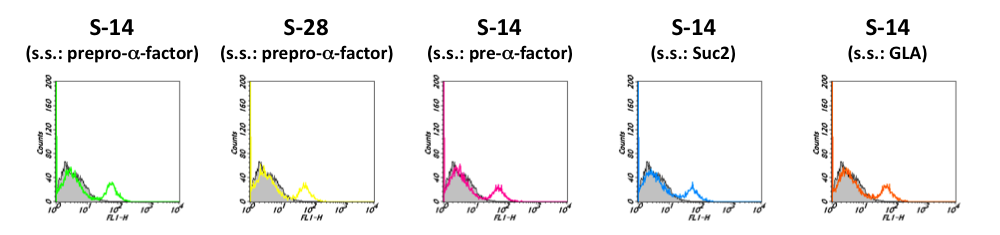

Supplement: Figure S3 — SSTR5 signaling assays of somatostatin-displaying yeast strains with various secretion signal sequences (color histograms). The Flo42 anchor was used for somatostatin display. S-28 indicates the 28 aa active isoform of somatostatin peptide. Gray histograms show the data from control strains (mock). Cultures were grown in SDM71 media for 22 h. IMG-50 was used as the host strain. The transformants used in this experiment are listed in Table S3. (TIF) [file pone.0037136.s003.tif]

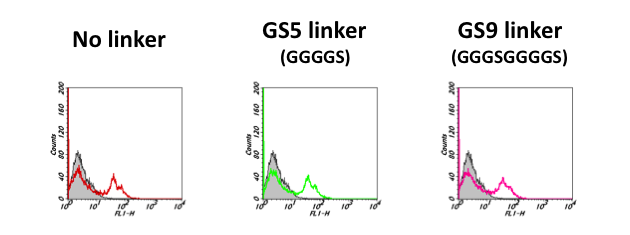

Supplement: Figure S4 — SSTR5 signaling assays of somatostatin-displaying yeast strains with different length GS linkers (color histograms). The S-14 peptide and Flo42 anchor were used for display. Gray histograms show the data from control strains (mock). Cultures were grown in SDM71 media for 12 h. IMG-50 was used as the host strain. The transformants used in this experiment are listed in Table S3. (TIF) [file pone.0037136.s004.tif]

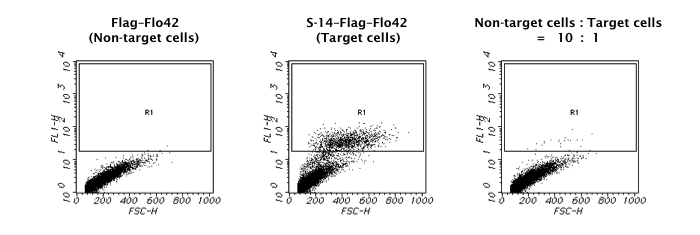

Supplement: Figure S5 — SSTR5 signaling assays of somatostatin-displaying yeast strain (target cells) mixed with somatostatin-non-displaying strain (non-target cells). S-14–Flag–Flo42 and Flag–Flo42 fusion proteins were used as target and non-target cells, respectively. R1 regions in the dot plots show the gates for FACS sorting. The ratio of initial cell densities was adjusted to 10∶1 (non-target cells : target cells), and the cultures were grown in SDM71 media. IMG-50 was used as the host strain. The transformants used in this experiment are listed in Table S3. (TIF) [file pone.0037136.s005.tif]
