# Supplementary material for: Cell Wall Trapping of Autocrine Peptides for Human G-Protein-Coupled Receptors on the Yeast Cell Surface
Source: PLoS One. 2012 May 18;7(5):e37136. doi: 10.1371/journal.pone.0037136 (PMC3356411; doi:10.1371/journal.pone.0037136)
Supplement: Table S1 — List of primers. (PDF) [file pone.0037136.s006.pdf]

Supporting Information:

Cell wall trapping of autocrine peptides for human G-protein-coupled receptors on the yeast cell surface

Table S1. List of primers

Construction of yeast strain with *bar1*Δ allele (IM-4)

Amplification of LEU2 marker from pRS405 (American Type Culture Collection, Manassas, VA, USA)

5’ATCGCCTAAAATCATACCAAAATAAAAAGAGTGTCTAGAAGGGTCATATATCGACTACGT CGTAAGGCCGTTTCTGACAG

5’ACTATATATTTGATATTTATATGCTATAAAGAAATTGTACTCCAGATTTCTCGACGGTCGA GGAGAACTTCTAGTATATC

Construction of α-factor secretion plasmid

Generation of s.s.-α-factor-FLAG-taa fragment for pUESCαsf from pUESCαf

5’-CCCCGGATCCATGAGATTTCCTTCAATTTT

5’-AAAACTCGAGTTACTTGTCATCGTCATCCT

Construction of α-factor displaying plasmids (Flo42, Flo102, Flo146, Flo318 and 3’AG)

Generation of s.s.-α-factor-FLAG fragment for pUESCαf from BY4741 genomic DNA

5’-CCCCGGATCCATGAGATTTCCTTCAATTTT

5’-AAAACTCGAGCTTGTCATCGTCATCCTTGTAGTCGTACATTGGTTGGCCAGGTTTTAGT TGCAACCAATGCCA

Generation of s.s.-α-factor-FLAG(+gg) fragment for pUESCαf(AG) from pUESCαf

5’-CCCCGGATCCATGAGATTTCCTTCAATTTT

5’-AAAACTCGAGCCCTTGTCATCGTCATCCTTGTAGTCGTACATTGGTTGGCCAGGTTTTA GTTGCAACCAATGCCA

Generation of FLO42 anchor fragment for pUESCαf-FLO42 from pIF42 (Sato et al.)

5’-TTCCCTCGAGAGCAGCATGGTAGGATATAG

5’-CCCCGCTAGCTTAAATAATTGCCAGCAATA

Generation of FLO102 anchor fragment for pUESCαf-FLO102 from pIF102 (Sato et al.)

5’-TGGGCTCGAGAAAACAGTAGTCACCTCTTC

5’-CCCCGCTAGCTTAAATAATTGCCAGCAATA

Generation of FLO146 anchor fragment for pUESCαf-FLO146 from pIF146 (Sato et al.)

5’-TTTTCTCGAGGCGAATGAAGAGTCTGTCAG

5’-CCCCGCTAGCTTAAATAATTGCCAGCAATA

Generation of FLO318 anchor fragment for pUESCαf-FLO318 from pIF318 (Sato et al.)

5’-GTTTCTCGAGTTACCACCTGCTACCACTACAAAAACGAGCGAACAAACCA

5’-CCCCGCTAGCTTAAATAATTGCCAGCAATA

Generation of 3'-half of α-agglutinin anchor fragment for pUESCαf-AG from BY4741 genomic DNA
5'-GGGACTCGAGCGCCAAAAGCTCTTTTATCT
5'-TCCCGCTAGCTTAGAATAGCAGGTACGACA

Construction of somatostatin (S-14) displaying plasmid

Generation of FLAG-FLO42 fragment for pGK426-tgFLO42 from pUESCαf-FLO42
5'-CCCCGTCGACGACTACAAGGATGACGATGACAAGAGCAGCATGGTAGGAT
5'-GGGGAGATCTTTAAATAATTGCCAGCAATA

Generation of s.s. fragment with partial S-14 sequence for overlapping PCR from pUESCαf
5'-GGGGGCTAGCATGAGATTTCCTTCAATTTT
5'-ATTCTTGCAGCCAGCTCTTTTATCCAAAGA

Generation of s.s.-S-14 fragment for pGK-S1442 by overlapping PCR
5'-GGGGGCTAGCATGAGATTTCCTTCAATTTT
5'-AAAAGTCGACACAGGATGTGAAAGTCTTCCAGAAGAAATTCTTGCAGCCAGC

Construction of other peptides displaying control plasmids

Generation of s.s. fragment with partial α-factor sequence for overlapping PCR from pUESCαf
5'-GGGGGCTAGCATGAGATTTCCTTCAATTTT
5'-TTGCAACCAATGCCATCTTTTATCCAAAGA

Generation of s.s.-α-factor fragment for pGK-alpha42 by overlapping PCR
5'-GGGGGCTAGCATGAGATTTCCTTCAATTTT
5'-AAAAGTCGACGTACATTGGTTGGCCAGGTTTTAGTTGCAACCAATGCCA

Generation of s.s.-AII fragment for pGK-AII42 from pUESCαf
5'-GGGGGCTAGCATGAGATTTCCTTCAATTTT
5'-TTTTGTCGACGAAGGGGTGTATGTACACCCGGTCTCTTTTATCCAAAGATACCC

Generation of s.s.-ET1 fragment for pGK-ET142 from pUESCαf
5'-GGGGGCTAGCATGAGATTTCCTTCAATTTT
5'-TTTTGTCGACCCAAATGATGTCCAGGTGGCAGAAGTAGACACACTCTTTATCCATCAGG
GACGAGCAGGAGCATCTTTTATCCAAAGATACCC

Generation of s.s. fragment without peptide sequence for pGK42 from pUESCαf
5'-GGGGGCTAGCATGAGATTTCCTTCAATTTT
5'-GGGGGTCGACTCTTTTATCCAAAGATACCC

Construction of α-factor displaying plasmids (Suc2(N), Suc2(C), FS(N) and FS(C))

Generation of SUC2(N) anchor fragment for pUESCαf-SUC2(N) from pWGP3-SUC2 (Tanino et al.)

5'-TGGGCTCGAGATGACAAACGAAACTAGCGA
5'-CCCCGCTAGCCTATTTTACTTCCCTTACTT

Generation of SUC2(C)-FLAG-α-factor fragment for pUESC-SUC2fα(C) from pWGP3-SUC2 (Tanino et al.)

5'-GGGAGTCGACATGCTTTTGCAAGCTTTCCT
5'-CCCCGCTAGCTTAGTACATTGGTTGGCCAGGTTTTAGTTGCAACCAATGCCACTTGTCA TCGTCATCCTTGTAGTCTTTTACTTCCCTTACTTGGAACT

Generation of FS(N) anchor fragment for pUESCαf-FS(N) from pWIFS (Matsumoto et al.)

5'-TTTTCTCGAGGAGGCGTGCTTACCAGCAGG
5'-AAAACCGCGGTTAGGTGATTTGTCCTGA

Generation of FS(C)-FLAG-α-factor fragment for pUESC-FSfα(C) from pWIFS (Matsumoto et al.)

5'-AAAAGGATCCATGACAATGCCTCATCGCTA
5'-CCCCCTCGAGTTAGTACATTGGTTGGCCAGGTTTTAGTTGCAACCAATGCCACTTGTCA TCGTCATCCTTGTAGTCGGTGATTTGTCCTGAAGATGATG

Construction of other types of somatostatin displaying plasmids

Generation of s.s.(prepro-α-factor) fragment with partial S-28 sequence for overlapping PCR from pUESCαf

5'-GGGGGCTAGCATGAGATTTCCTTCAATTTT
5'-GTTTGAGTTAGCAGATCTTTTATCCAAAGA

Generation of s.s.(prepro-α-factor)-S-28 fragment for pGK-S2842 by overlapping PCR

5'-GGGGGCTAGCATGAGATTTCCTTCAATTTT
5'-AAAAGTCGACACAGGATGTGAAAGTCTTCCAGAAGAAATTCTTGCAGCCAGCTTTGCGT TCTCGGGGTGCCATAGCCGGGTTTGAGTTAGCAGA

Generation of s.s.(pre-α-factor)-S-14 fragment for pGK-AS1442 with synthetic oligonucleotides

5'-AAAAGCTAGCATGAGATTTCCTTCAATTTTTACTGCAGTTTTATTCGCAGCATCCTCCGC ATTAGCTGCTGGCTGCAAGAATTTCTTCTGGAAGACTTTCACATCCTGT
5'-AAAAGTCGACACAGGATGTGAAAGTCTTCCAGAAGAAATTCTTGCAGCCAGC

Generation of s.s.(Suc2)-S-14 fragment for pGK-SS1442 with synthetic oligonucleotides

5'-AAAAGCTAGCATGCTTTTGCAAGCTTTCCTTTTCCTTTTGGCTGGTTTTGCAGCCAAAAT ATCTGCAGCTGGCTGCAAGAATTTCTTCTGGAAGACTTTCACATCCTGT
5'-AAAAGTCGACACAGGATGTGAAAGTCTTCCAGAAGAAATTCTTGCAGCCAGC

Generation of s.s.(GLA)-S-14 fragment for pGK-GS1442 with synthetic oligonucleotides
5’-AAAAGCTAGCATGCAACTGTTCAATTTGCCATTGAAAGTTTCATTCTTTCTCGTCCTCTC TTACTTTTCTTTGCTCGTTTCTGCTGCTGGCTGCAAGAATTTCTTCTGGAAGACTTTCAC
5’-AAAAGTCGACACAGGATGTGAAAGTCTTCCAGAAGAAATTCTTGCAGCCAGC

Generation of s.s.(prepro-α-factor)-S-14-GS5 (GGGGS linker) fragment for pGS5-S1442 from pGK-S1442
5’-GGGGGCTAGCATGAGATTTCCTTCAATTTT
5’-TTTTGTCGACAGAACCACCACCACCACAGGATGTGAAAGTCTTCCAGAAG

Generation of s.s.(prepro-α-factor)-S-14-GS9 (GGGSGGGGS linker) fragment for pGS9-S1442 from pGK-S1442
5’-GGGGGCTAGCATGAGATTTCCTTCAATTTT
5’-TTTTGTCGACAGAACCACCACCACCAGAACCACCACCACAGGATGTGAAAGTCTTCCA GAAGAAATTCTTGCAGCC
