# Supplementary material for: Cell Wall Trapping of Autocrine Peptides for Human G-Protein-Coupled Receptors on the Yeast Cell Surface
Source: PLoS One. 2012 May 18;7(5):e37136. doi: 10.1371/journal.pone.0037136 (PMC3356411; doi:10.1371/journal.pone.0037136)
Supplement: Table S2 — Plasmids used in Supplementary data. (PDF) [file pone.0037136.s007.pdf]

Table S2. Plasmids used in Supplementary data

Plasmid	Relative feature	Source
**α-factor display**		
pUESCαf-SUC2(N)	pESC-URA, α-factor–Flag–Suc2(N) fusion protein expression (for display)	This study
pUESC-SUC2fα(C)	pESC-URA, Suc2(C)–Flag–α-factor fusion protein expression (for display)	This study
pUESCαf-FS(N)	pESC-URA, α-factor–Flag–FS(N) fusion protein expression (for display)	This study
pUESC-FSfα(C)	pESC-URA, FS(C)–Flag–α-factor fusion protein expression (for display)	This study
**Somatostatin display**		
pGK-S2842	pGK426, s.s.(prepro-α-factor)–S-28–Flag–Flo42[a] fusion protein expression (for display)	This study
pGK-AS1442	pGK426, s.s.(pre-α-factor)–S-14–Flag–Flo42 fusion protein expression (for display)	This study
pGK-SS1442	pGK426, s.s.(Suc2)–S-14–Flag–Flo42[b] fusion protein expression (for display)	This study
pGK-GS1442	pGK426, s.s.(GLA)–S-14–Flag–Flo42[c] fusion protein expression (for display)	This study
pGS5-S1442	pGK426, s.s.(prepro-α-factor)–S-14–GS5–Flag–Flo42[d] fusion protein expression (for display)	This study
pGS9-S1442	pGK426, s.s.(prepro-α-factor)–S-14–GS9–Flag–Flo42[e] fusion protein expression (for display)	This study

All transcription products without instructions contain the secretion signal sequences of prepro-α-factor.
[a]S-28 encodes 28 aa peptide of somatostatin active isoform.
[b]Suc2 signal sequence is derived from *S. cerevisiae*.
[c]GLA indicates signal sequence of glucoamylase derived from *R. oryzae*.
[d]GS5 indicates GGGGS linker.
[e]GS9 indicates GGGSGGGGS linker.
