# Supplementary material for: Cell Wall Trapping of Autocrine Peptides for Human G-Protein-Coupled Receptors on the Yeast Cell Surface
Source: PLoS One. 2012 May 18;7(5):e37136. doi: 10.1371/journal.pone.0037136 (PMC3356411; doi:10.1371/journal.pone.0037136)
Supplement: Table S3 — List of strains and transformants used for assays. (PDF) [file pone.0037136.s008.pdf]

Cell wall trapping of autocrine peptides for human G-protein-coupled receptors on the yeast cell surface

Table S3. List of strains and transformants used for assays

Strain/plasmid	Description
**Figure 3**	
IMG-4/pESC-URA	Mock control
IMG-4/pUESCαsf	α-factor secretion
IMG-4/pUESCαf-AG	α-factor display by AG anchor
IMG-4/pUESCαf-FLO42	α-factor display by Flo42 anchor
IMG-4/pUESCαf-FLO102	α-factor display by Flo102 anchor
IMG-4/pUESCαf-FLO146	α-factor display by Flo146 anchor
IMG-4/pUESCαf-FLO318	α-factor display by Flo318 anchor
**Figure 4A, 5 and 7**	
IMFD-70/pGK421/pGK426	Mock control / Mock control
IMFD-70/pGK-SSTR5-HA/pGK426	SSTR5 expression / Mock control
IMFD-70/pGK-SSTR5-HA/pGK42	SSTR5 expression / Flo42 anchor display (no peptide)
IMFD-70/pGK-SSTR5-HA/pGK-S1442	SSTR5 expression / S-14 display by Flo42 anchor
IMFD-70/pGK421/pGK-S1442	Mock control / S-14 display by Flo42 anchor
**Figure 4B**	
IMFD-70/pGK-SSTR5-HA/pGK-alpha42	SSTR5 expression / α-factor display by Flo42 anchor
IMFD-70/pGK-SSTR5-HA/pGK-AII42	SSTR5 expression / AII display by Flo42 anchor
IMFD-70/pGK-SSTR5-HA/pGK-ET142	SSTR5 expression / ET1 display by Flo42 anchor
**Figure 6**	
IMFD-70/pGK-SSTR5-HA/pGK42 /pMHG-FIG1	SSTR5 expression / Flo42 anchor display (no peptide)
IMFD-70/pGK-SSTR5-HA/pGK-S1442 /pMHG-FIG1	SSTR5 expression / S-14 display by Flo42 anchor
**Figure S1**	
IMG-4/pUESCαf-FLO42	α-factor display by Flo42 anchor
**Figure S2**	
IMG-4/pESC-URA	Mock control
IMG-4/pUESCαf-FLO42	α-factor display by Flo42 anchor
IMG-4/pUESCαf-SUC2(N)	α-factor display by Suc2(N) anchor
IMG-4/pUESCαf-SUC2(C)	α-factor display by Suc2(C) anchor
IMG-4/pUESCαf-FS(N)	α-factor display by FS(N) anchor
IMG-4/pUESCαf-FS(C)	α-factor display by FS(C) anchor
**Figure S3**	
IMG-50/pGK-SSTR5-HA/pGK42	SSTR5 expression / Mock control
IMG-50/pGK-SSTR5-HA/pGK-S1442	SSTR5 expression / S-14 display by Flo42 anchor (s.s. of prepro-α-factor)
IMG-50/pGK-SSTR5-HA/pGK-S2842	SSTR5 expression / S-28 display by Flo42 anchor (s.s. of prepro-α-factor)
IMG-50/pGK-SSTR5-HA/pGK-AS1442	SSTR5 expression / S-14 display by Flo42 anchor (s.s. of pre-α-factor)
IMG-50/pGK-SSTR5-HA/pGK-SS1442	SSTR5 expression / S-14 display by Flo42 anchor (s.s. of Suc2)
IMG-50/pGK-SSTR5-HA/pGK-GS1442	SSTR5 expression / S-14 display by Flo42 anchor (s.s. of GLA)
**Figure S4**	
IMG-50/pGK-SSTR5-HA/pGK42	SSTR5 expression / Mock control
IMG-50/pGK-SSTR5-HA/pGK-S1442	SSTR5 expression / S-14 display by Flo42 anchor
IMG-50/pGK-SSTR5-HA/pGK-S1442	SSTR5 expression / S-14 display by Flo42 anchor (inserting GS5 linker)
IMG-50/pGK-SSTR5-HA/pGS5-S1442	SSTR5 expression / S-14 display by Flo42 anchor (inserting GS9 linker)
**Figure S5**	
IMG-50/pGK-SSTR5-HA/pGK42	SSTR5 expression / Mock control
IMG-50/pGK-SSTR5-HA/pGK-AS1442	SSTR5 expression / S-14 display by Flo42 anchor (s.s. of pre-α-factor)
