# Supplementary material for: Cell Wall Trapping of Autocrine Peptides for Human G-Protein-Coupled Receptors on the Yeast Cell Surface
Source: PLoS One. 2012 May 18;7(5):e37136. doi: 10.1371/journal.pone.0037136 (PMC3356411; doi:10.1371/journal.pone.0037136)
Supplement: Document S1 — Supplementary Materials and Methods (Plasmid constructions for supporting information). (PDF) [file pone.0037136.s009.pdf]

Document S1. Supplementary Materials and Methods (Plasmid constructions for supporting information)

All plasmids used for supplementary data are listed in Table S2. All primers used for plasmid constructions are listed in Table S1. The amplified gene encoding Suc2(N) anchor was inserted at XhoI and NheI sites into pUESCαf, resulting in pUESCαf-SUC2(N). pUESCαf-FS(N) was produced in a similar procedure by inserting the gene encoding FS(N) anchor at XhoI and SacII sites into pUESCαf. The genes encoding Suc2(C) anchor or FS(C) anchor, FLAG-tag and α-factor mature peptide were inserted at SalI and NheI sites or BamHI and XhoI sites into pESC-URA to create pUESC-SUC2fα(C) or pUESC-FSfα(C). The DNA fragment containing s.s. of α-factor (prepro-α-factor) and S-28 mature peptide was amplified by overlapping PCR and inserted at NheI and SalI sites into pGK426-tgFLO42, producing pGK-S2842. The DNA fragments containing three types of s.s. (pre-α-factor signal sequence, Suc2 signal sequence and GLA signal sequence; glucoamylase signal sequence derived from *Rhizopus oryzae*) and S-14 mature peptide were prepared by annealing synthetic oligonucleotides and inserted at NheI and SalI sites into pGK426-tgFLO42, generating pGK-AS1442, pGK-SS1442 and pGK-GS1442, respectively. The amplified DNA fragment containing s.s. (prepro-α-factor), S-14 mature peptide and GS linker (GS5, GGGGS linker; or GS9, GGGSGGGGS linker) was inserted at NheI and SalI sites into pGK426-tgFLO42, obtaining pGS5-S1442 or pGS9-S1442. The transformation procedure of plasmids followed the lithium acetate method. All transformants used for assays are listed in Table S3.
